# The role of preclinical SPECT in oncological and neurological research in combination with either CT or MRI

**DOI:** 10.1007/s00259-013-2685-3

**Published:** 2014-04-17

**Authors:** Monique R. Bernsen, Pieter E. B. Vaissier, Roel Van Holen, Jan Booij, Freek J. Beekman, Marion de Jong

**Affiliations:** 1 Department of Nuclear Medicine, Erasmus MC, Rotterdam, The Netherlands; 2 Department of Radiology, Erasmus MC, Rotterdam, The Netherlands; 3 Section Radiation Detection and Medical Imaging, Delft University of Technology, Delft, The Netherlands; 4 ELIS Department, MEDISIP, Ghent University, iMinds, Ghent, Belgium; 5 Department of Nuclear Medicine, Academic Medical Center, University of Amsterdam, Amsterdam, The Netherlands; 6 MILabs B.V., Utrecht, The Netherlands

## Abstract

Preclinical imaging with SPECT combined with CT or MRI is used more and more frequently and has proven to be very useful in translational research. In this article, an overview of current preclinical research applications and trends of SPECT combined with CT or MRI, mainly in tumour imaging and neuroscience imaging, is given and the advantages and disadvantages of the different approaches are described. Today SPECT and CT systems are often integrated into a single device (commonly called a SPECT/CT system), whereas at present combined SPECT and MRI is almost always carried out with separate systems and fiducial markers to combine the separately acquired images. While preclinical SPECT/CT is most widely applied in oncology research, SPECT combined with MRI (SPECT/MRI when integrated in one system) offers the potential for both neuroscience applications and oncological applications. Today CT and MRI are still mainly used to localize radiotracer binding and to improve SPECT quantification, although both CT and MRI have additional potential. Future technology developments may include fast sequential or simultaneous acquisition of (dynamic) multimodality data, spectroscopy, fMRI along with high-resolution anatomic MRI, advanced CT procedures, and combinations of more than two modalities such as combinations of SPECT, PET, MRI and CT all together. This will all strongly depend on new technologies. With further advances in biology and chemistry for imaging molecular targets and (patho)physiological processes in vivo, the introduction of new imaging procedures and promising new radiopharmaceuticals in clinical practice may be accelerated.

## Introduction

Over the past decade the use of PET, SPECT, CT and MRI in preclinical research has greatly increased due to technological advances that have resulted in significant improvements in spatial and temporal resolution as well as sensitivity [1–5]. These noninvasive imaging methods enable imaging of (patho)physiological and molecular processes over time in vivo, obviating the need for killing animals for each time-point being studied [6–8]. Each of these imaging modalities has unique qualities, in terms of their spatial and temporal resolution and their ability to measure morphology and/or function; the appropriate technique should be selected according to the research question. PET and SPECT allow detection of radiopharmaceuticals at nano- to picomolar concentrations in vivo, and have proven to be excellent tools in the translational evaluation of radiotracers. CT and MRI provide a high degree of spatial resolution that is well suited to anatomical imaging and tissue phenotyping, including volumetry, and can provide information regarding tissue physiology [9].

Due to their sensitive detection capabilities, PET and SPECT both have preeminent ability to monitor and quantify dynamic processes at a molecular level in vivo. Unique SPECT capabilities include: the ability to image ligands such as peptides and antibodies relatively easy with ^99m^Tc, ^111^In or iodine isotopes (^123^I, ^125^I), the ability to measure slow kinetic processes due to the long half-life (compared to most PET tracers) of some of the commonly used radionuclides, and the ability to probe multiple molecular pathways simultaneously by detecting radionuclides with different gamma energies (multiisotope imaging). Multiisotope imaging has been demonstrated both clinically [10–13] and preclinically [14, 15]. Another advantage of SPECT over PET is that no cyclotron and associated infrastructure and complex logistics are required on site and that many tracers are readily available in the form of kits.

**Fig. 1:**
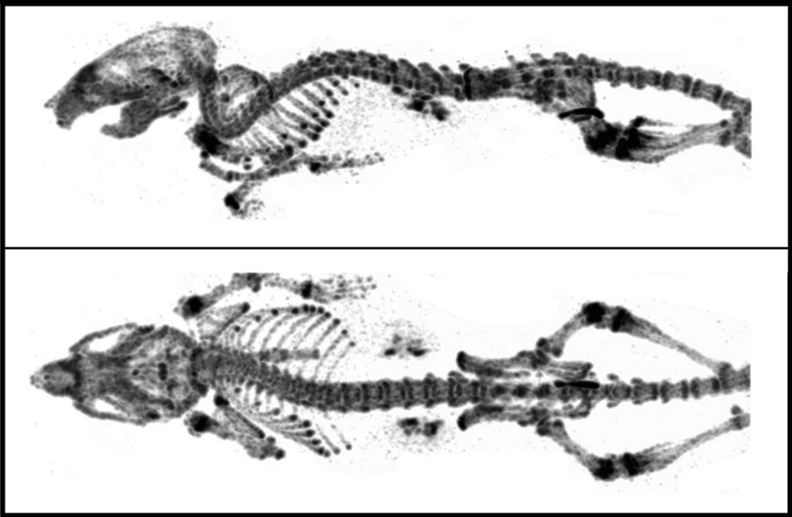
State-of-the-art whole-body SPECT bone images acquired for 60 min with 250 MBq ^99m^Tc-HDP and with 0.25-mm resolution collimators (image courtesy of Oleksandra Ivashchenko, TU-Delft/MIlabs)

While in clinical imaging higher spatial resolutions can be obtained with PET than with SPECT, the opposite is clearly true in preclinical imaging in small animals. Small imaging volumes enable the use of high magnification apertures in SPECT imaging (Fig. 1), increasing sensitivity and resolution relative to their clinical counterparts [16–18]. Recently developed SPECT systems can be extended to high-resolution imaging of high-energy photons emitted by PET tracers, even simultaneously with (multiple) SPECT tracers [14]. Since some SPECT systems also enable imaging of ^125^I-labelled tracers (<35 keV), the gap between in vitro and in vivo studies is closed. Finally, in SPECT imaging spatial resolution and sensitivity can be adjusted by changing the size of the collimator apertures.

On the other hand, the drawbacks of SPECT include its lower sensitivity compared to PET, especially when high-resolution SPECT is desired. Moreover, SPECT tracer molecules may differ with regard to their biological properties from their nonradioactive counterparts after introduction of a radionuclide–chelator complex, which is not the case for several PET tracers in which endogenous atoms (such as hydrogen, carbon and oxygen) can be replaced by their radioactive isotopes. In addition, the dynamic capabilities of SPECT, although recently greatly improved, are often limited compared to those of PET.

In current clinical practice combining images from different tomographic modalities is common. Also in preclinical research multimodality imaging strategies are useful, as different modalities can provide highly complementary information. Spatially registered images enable localization, enhanced visualization and accurate quantification of spread and uptake of radiolabelled molecules within the anatomical context provided by CT or MRI. In addition, functional information derived from advanced CT and MRI techniques such as perfusion imaging can be related to expression and function of specific molecules as measured by PET or SPECT.

In this review we discuss recent applications and technological advances of preclinical SPECT in combination with CT or MRI in the fields of oncology and neuroscience. Overviews by others and Golestani et al. addressing preclinical SPECT combined with MRI and CT in other research fields, such as cardiovascular research, regenerative medicine and inflammation, have recently been published [19–22]. The space constraints of this article prevented coverage of every aspect of this exciting field, but we aimed to provide a good appreciation of the possibilities, and also the limitations and remaining challenges.

## Applications of SPECT combined with CT or MRI

### Tumour imaging

Hanahan and Weinberg [23, 24] introduced the notion that the tumour microenvironment plays a crucial role in the development and behaviour of tumours, including receptiveness and sensitivity to treatment. The resulting understanding that cancer is a complex disease with significant involvement of the tumour stroma has led to the interest in imaging tumour cell characteristics as well as noncancer cell components in vivo [25, 26], especially with regard to molecular diagnostics and drug development. Since it would be impossible to cover every aspect of this rapidly developing field, we only address some key aspects in tumour imaging and the roles that SPECT, and SPECT combined with CT or MRI have been playing in this field.

#### Imaging targets and probes

Tumours and tumour cells exhibit different characteristics compared to normal tissue and cells; this is reflected in altered physiology, tissue composition and expression of intra- and extracellular molecules [23, 24, 26–28]. All these aspects can be used as imaging targets in relation to diagnostics, drug development and treatment response assessment. SPECT probes (or tracers) can be classified according to their biodistribution and targeting characteristics, i.e. the biodistribution of some radiopharmaceuticals is determined by their chemical/physical properties, whereas that of other tracers is determined by their specific interaction with a target. For details the reader is referred to a review by Müller and Schibli [29].

Tumours are known often to display an aberrant vascular network and microcirculation, which in turn underlies features such as interstitial hypertension, hypoxia and acidosis, characteristics that contribute to malignant phenotypes and resistance to various treatments [30]. Within this environment, tumour cells can also display altered energy metabolism, as reflected in, for example, increased glucose uptake and shifted balances in metabolic products. At the preclinical level, a variety of SPECT tracers are under evaluation for use as markers for (neo)angiogenesis [31–33], hypoxia [34–37], acidosis [38–40], metabolic activity [41] and proteolytic activity [42, 43]. Moreover, MRI and to a lesser extend CT offer options for interrogating tumour physiological characteristics, either through the use of specific probes or the use of sophisticated MRI techniques, as recently reviewed by Bernsen et al. [9]. Besides metabolic tracers, much effort has been put into the development and validation of SPECT probes specific for tumour target molecules such as antigens, receptors or other molecules also overexpressed in tumour tissue. The use of peptides interacting with receptors [44], antibodies and antibody fragments targeting their epitopes [45], vitamin-based radiopharmaceuticals [28] and nucleoside analogues [46], significantly increases the possibilities for tumour detection, localization and staging.

Specific points of interest in translational preclinical imaging studies include efforts directed at improved tumour specificity [47], tumour uptake/retention [48] and minimized pharmacological effects [49, 50] of imaging probes. In most preclinical studies involving the use of SPECT combined with CT or MRI to date, the CT or MRI components have been mostly used to provide anatomical reference and more recently also for attenuation correction [51]. However, CT and MRI offer more than anatomical information, and some examples of the use of more sophisticated CT and MRI techniques are discussed and provided in the technology sections below.

#### Biodistribution studies/dosimetry/response assessment

In drug development, biodistribution and pharmacokinetic properties of a candidate drug or therapeutic agent are crucial for their therapeutic potential and safety in patients. After binding of a suitable radionuclide to the molecule or particle of interest, preclinical SPECT imaging provides a valuable noninvasive tool to study candidate drugs. Especially in development of targeted treatment strategies with radiolabelled molecules such as peptides, antibodies and vitamin-based analogues, SPECT imaging combined with CT or MRI has been widely used [45, 52–56]. Next to in vivo evaluation of such molecules, SPECT combined with CT or MRI is also being applied in the preclinical evaluation of (nano)particles for treatment and/or diagnosis of cancer. Various studies have investigated the biodistribution and therapeutic potential of, for example, liposomes [57–61], radiolabelled superparamagnetic iron oxide nanoparticles and ^166^Ho microspheres (^166^HoAcAcMS), using multimodality imaging approaches with SPECT/CT and SPECT/MRI [62, 63]. The combined imaging data allow accurate assessment of biodistribution and retention as well as dosimetry calculations.

Many of the imaging biomarkers addressed in the previous section are also being evaluated as markers to monitor response to treatment. Elimination of tumour cells might be accompanied by loss of tracer uptake directed at tumour-associated antigens or decreased metabolic activity, whereas changes in vascular properties and tissue hypoxia may be expected after antiangiogenic therapies, allowing these markers to be used for response assessment. While such an approach may appear fairly straightforward, some limitations and pitfalls need to be taken into account. Loss of tumour-associated antigen expression may also be a result of changed tumour physiology not related to tumour cell death [64]. Another process of interest as an imaging biomarker for response is apoptosis [65, 66]. Expectations were raised that visualization and quantification of apoptosis, as a more specific and relevant marker of cell death, may provide better specificity for assessing actual tumour cell elimination following treatment. Apoptosis imaging using a tracer specific for annexin could reveal early tumour cell death after chemotherapy [65], but its value as a robust marker for treatment response still needs to be established.

**Fig. 2:**
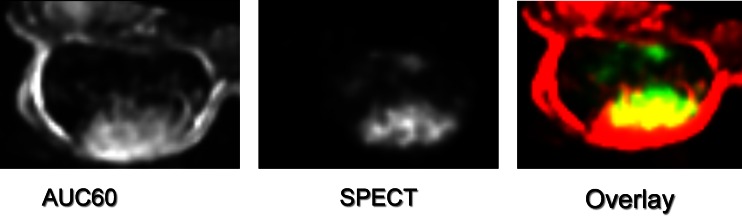
Multimodality imaging of tumour uptake of targeted radiolabelled peptide and tumour perfusion. Rats bearing a syngeneic, somatostatin receptor overexpressing, neuroendocrine pancreatic tumour, were imaged by SPECT/CT and MRI to study tumour uptake of a ^111^In-labelled somatostatin analogue ([^111^In-DTPA]octreotide) and tumour perfusion by DCE-MRI respectively. *Left* Tumour perfusion depicted by the AUC value over the first 60 s as assessed by DCE-MRI; *centre* tumour uptake of radiolabelled [^111^In-DTPA]octreotide of the same tumour section as imaged by MRI; *right* colour-coded overlay of the MR image and the SPECT image with MRI values depicted in *red* and SPECT values depicted in *green*. For correct image registration, MRI data were resampled to match the lower resolution of the SPECT/CT images (image courtesy of Joost Haeck and Karin Bol, Erasmus MC)

For the assessment of potential treatment efficacy, Bol et al. recently reported on the added value of dual modality imaging using SPECT and MRI [67]. In a rat model of neuroendocrine pancreatic tumour, radiolabelled peptide uptake was assessed in conjunction with measurement of tumour perfusion using DCE-MRI. A substantial correlation between tumour uptake of ^111^In-DTPA-octreotide and tumour perfusion parameters was observed (Fig. 2). It was shown that even in tumour areas with high receptor expression no peptide uptake occurred when perfusion was low, indicating that combined SPECT and MRI may be useful in treatment planning and/or response prediction in patients treated with PRRT.

Imaging of cell trafficking has also been an area of interest in which SPECT in combination with either CT or MRI has been employed, an approach that has already been part of clinical routine for several decades for identifying infection or inflammation sites by leucocyte scintigraphy [68]. Recently, the interest in in vivo cell tracking has received a tremendous boost from the realization that knowledge about the in vivo fate of infused cells is crucial to the development of safe and effective cell-based therapeutic strategies, including stem cell therapy [69, 70]. SPECT has largely been used to investigate the short-term fate of transplanted cells labelled with radiotracers such as ^111^In-oxine, ^99m^Tc-hexamethylpropylene amine oxine (HMPAO) and ^111^In-tropolone as intracellular labels [71]. However, due to the lack of anatomical information and the limited life-time of the radionuclides, preventing longitudinal follow up, other imaging techniques such as MRI have been widely used as well [72]. Since MRI also has some specific limitations for in vivo cell tracking such as low sensitivity and specificity, and challenges in quantification of the MRI probe, alternative approaches have been sought, with specific interest in reporter gene technology [70]. For SPECT the sodium iodide symporter gene (NIS) and the herpes simplex virus type 1 thymidine kinase gene (HSV1-tk) are so far the most commonly used reporter genes in combination with radioactive substrates [73, 74]. Reporter gene technology with these and other reporter genes, e.g. norepinephrine transporter and the somatostatin receptor, is being used not only in in vivo cell tracking applications for cell-based therapy [75, 76], but also to monitor metastatic spread of tumour cells [77–79], as well as gene delivery and expression of genes in targeted gene therapy approaches [80, 81].

Finally, in medical research, the successful choice of a target molecule that is a key disease biomarker has the potential to lead to the development not only of a molecular imaging probe, but also of a therapeutic agent to inhibit the disease process. Examples include peptides [53, 82, 83], antibodies or fragments thereof [84–87], and nanoparticles [26, 88], similar compounds or particles that can be labelled with radionuclides for either imaging or therapy. Receptor targeting with small radiolabelled peptides for receptor-targeted tumour imaging (PET and SPECT) as well as for radionuclide therapy [89] provide good examples of such theranostic potential in nuclear oncology and have paved the way for further developments in this field.

### Neuroscience

#### Preclinical SPECT studies in small laboratory animal models of neurodegenerative diseases

Parkinson’s disease (PD) is a neurodegenerative disease characterized by loss of neurons producing dopamine (DA), and consequently loss of the DA transporter (DAT) [90–95]. Preclinical SPECT studies initially focused on the feasibility of detecting striatal DAT binding in small laboratory animals per se [96, 97]. In the past decade, pinhole SPECT studies have shown the possibility of detecting loss of striatal DAT binding in rodent models of PD using [^123^I]FP-CIT and [^123^I]β-CIT as radiotracers [98, 99]. Initially, single-pinhole SPECT systems were used to image DAT [91, 100], and the SPECT images were coaligned with MR images (or templates) acquired on clinical MRI scanners (using dedicated coils), with or without the use of external markers [99, 101, 102]. Another recent study, however, used a preclinical system with high-resolution parallel-hole collimators (X-SPECT system) to evaluate DAT loss (using [^123^I]altropane as a radiotracer) in a rat model of PD, and the SPECT images were registered with CT images [103]. Another DAT ([^123^I]FP-CIT) SPECT study in a mouse model of PD used a double-headed gamma camera equipped with a multipinhole aperture. The SPECT images were not coaligned with CT or MR images [104, 105]. Finally, MRI is an important tool in the field of neuroimaging. In this regard, it is of interest that Lee et al. proposed an image registration algorithm which can be used to register individual DAT SPECT ([^99m^Tc]TRODAT was used as a radiotracer on a NanoSPECT/CT system) and brain MR images (acquired on a 3-T system) in rodent models of PD without using external markers [106].

Neurodegenerative diseases like multiple system atrophy, progressive supranuclear palsy and Huntington’s disease, are characterized by loss of striatal DA D_2_ receptors [92]. A study published in 2002 demonstrated the feasibility of pinhole SPECT for measuring striatal DA D_2/3_ receptor binding in the mouse brain in vivo [107]. [^123^I]IBF was used to assess striatal D_2/3_ receptor binding and SPECT images were not registered with CT or MR images. Not long afterwards, another study in rats confirmed the feasibility of assessing DA D_2/3_ receptor binding in- vivo, using [^123^I]IBZM as radiotracer and a dedicated small animal SPECT system [108]. In that study, SPECT images were not registered with CT or MR images, but a region of interest template was constructed and used to evaluate receptor binding [108].

Scherfler et al. showed the ability of single-pinhole SPECT to detect loss of striatal DA D_2/3_ receptors in a rat model of Huntington’s disease [109]. In that study, the [^123^I]IBZM SPECT images were registered on a MRI template. Importantly, in vivo [^123^I]IBZM binding was highly correlated with the loss of medium-sized spiny neurons that express DA D_2_ receptors demonstrated ex vivo [110].

Alzheimer’s disease (AD) is the most common dementia in humans, and is characterized by the deposition of β-amyloid plaques and neurofibrillary tangles. PET tracers have been developed successfully to image this neuropathology [111]. The deposition of amyloid has also been evaluated in micro-PET studies in animal models of AD [112, 113]. SPECT tracers have also been developed for labelling of amyloid plaques [112]. Although [^123^I]IMPY shows high affinity for amyloid in vitro and amyloid plaques in post-mortem brain tissue of AD patients and animal models of AD, the specific to nonspecific binding ratios are too low to be of value for studies in animal models of AD [114, 115].

#### Preclinical SPECT studies in small laboratory animals relevant to studies on psychosis or addiction

A consistent finding of imaging studies in drug addiction is loss of striatal DA D_2/3_ receptors. An increase in D_2_ receptor expression may therefore be beneficial in its treatment [116]. Interestingly, some drugs may induce an increase in D_2/3_ receptors [117–119], which has been supported by SPECT imaging in rats [117]. In the latter study an ultrahigh-resolution pinhole SPECT system was used (U-SPECT-II), but SPECT images were not registered with CT or MR images. Due to the high spatial as well as temporal resolution of this system, changes in DAT occupancy by cocaine over time can be studied in the mouse in vivo [120]. Alterations in the expression of DA D_2/3_ receptors have been reported in schizophrenia. In a recent study, in which the SPECT images were registered with CT images (X-SPECT/CT system), decreases in DA D_2/3_ receptor availability in the striatum and midbrain have been shown in a rat model of schizophrenia using [^123^I]epidepride as radiotracer [121]. DA D_2/3_ receptor imaging can be used to evaluate DA release [122]. Increased DA release has been reported in schizophrenia, whereas DA release may be reduced in cocaine dependency [123, 124]. Interestingly, recent pinhole SPECT studies in mice and rats have also shown the ability to measure DA release [125, 126]. In both studies, SPECT images were not registered with CT or MR images.

#### Preclinical SPECT studies focused on brain perfusion

Brain perfusion studies may be of relevance for the study of, for example, the aetiology of stroke. Using a multipinhole SPECT system (NanoSPECT), the kinetics of the perfusion tracers [^99m^Tc]HMPAO and [^99m^Tc]ECD were compared directly in control mice. SPECT images were registered on a MRI template [127]. It was shown that [^99m^Tc]ECD washout was much faster than that of [^99m^Tc]HMPAO. In another study, [^123^I]iodoamphetamine was used to assess hypoperfusion in infarcted brain areas in mice [128]. A single-pinhole collimator system was used, and CT and MRI images acquired on other systems were used for the alignment of the SPECT images. Finally, Ceulemans et al. performed brain perfusion SPECT studies ([^99mTc^]HMPAO, 1-mm pinhole collimator positioned on a dual-head gamma camera, coregistered on individual CT images) to quantify the infarct size in rats [129].

**Fig. 3:**
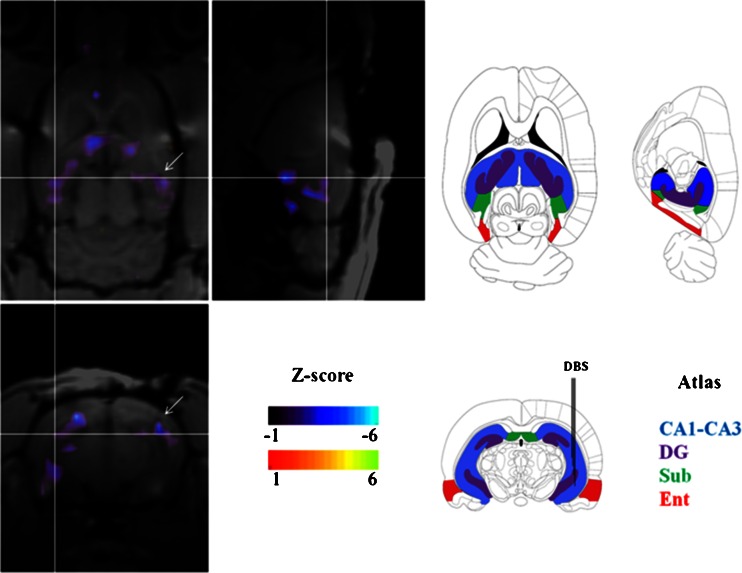
Coronal, sagittal and transverse anatomical T1-weighted MRI scans coregistered with coloured subtraction SPECT data illustrating the changes in regional cerebral blood flow induced by deep brain stimulation (*DBS*). The *white arrows* indicate a DBS electrode artefact in the hippocampus. The corresponding sections, modified from the rat brain atlas of Paxinos and Watson [183] are shown on the right (*CA1-CA3*; *DG* dentate gyrus, *Sub* subiculum, *Ent* entorhinal cortex). The different hippocampal structures are coloured and the position of the DBS electrode is indicated (courtesy Tine Wyckhuys [131])

Deep brain stimulation (DBS) is commonly used in the treatment of PD, but has recently also been used in the treatment of other neuropsychiatric disorders [130]. Interestingly, Wyckhuys et al. studied the effects of DBS on brain perfusion in rats [131]. In all rats, they acquired individual brain perfusion studies with SPECT (U-SPECT-II) after DBS (stimulator on and off), micro-CT scans and, after the animals were killed and the electrodes removed, MRI scans on a clinical MRI scanner using a dedicated rat brain oil [131]. After registration of the images and analysis of each voxel, hypoperfusion induced by DBS could be located accurately in small brain areas (Fig. 3). This approach highlights the potential of multimodality imaging to evaluate and locate the effects of interventions/treatments in small brain areas of rodents.

#### Preclinical SPECT studies focused on neurooncology

Micro-SPECT studies have also been performed successfully in the field of neurooncology. For example, Yang et al. recently showed the feasibility of using [^99m^Tc]DTPA to study the integrity of the blood–brain barrier and tumour activity in glioma-bearing rats [132]. A preclinical pinhole SPECT/CT system (FLEX Triumph) was used which offers the ability to coalign the SPECT and CT images [132]. Angiogenesis is essential for tumour growth. Furthermore, malignant cells can release vascular endothelial growth factors (VEGFs) which are important promoters and regulators of angiogenesis. SPECT studies showed the possibility of imaging VEGF receptors in rats. [^99m^Tc]HYNIC-VEGF uptake was increased in glioma-bearing rats pretreated with a VEGR receptor tyrosine kinase inhibitor [64]. In that study, SPECT images were acquired on a dedicated multiple-pinhole SPECT system (NanoSPECT), but the SPECT images were not registered with CT or MR images. In addition, Huang et al. evaluated a ^188^Re-labelled liposome as a diagnostic and therapeutic agent in glioma-bearing rats [60], using a preclinical multiple-pinhole SPECT/CT system (NanoSPECT/CT). Importantly, uptake in the brain tumour could be visualized, and specific binding was confirmed histopathologically [121]. Another study in glioma-bearing rats evaluated new treatment strategies for glioma, and imaged ^99m^Tc-labelled nanoparticles using a clinical SPECT system [133]. Finally, SPECT/CT (parallel hole SPECT system) studies were performed to examine successfully glioblastoma xenografts that were located subcutaneously in mice using, for example, ^125^I-labelled monoclonal antibodies against chemokine receptor 4 [134].

## Technology of SPECT combined with CT or MRI

### Combined imaging approaches/systems, introduction

In order to fully benefit from multimodality imaging, accurate spatial registration of the images is crucial. Below we address ways to adequately combine SPECT with CT or MRI.

#### Side-by-side systems

**Fig. 4:**
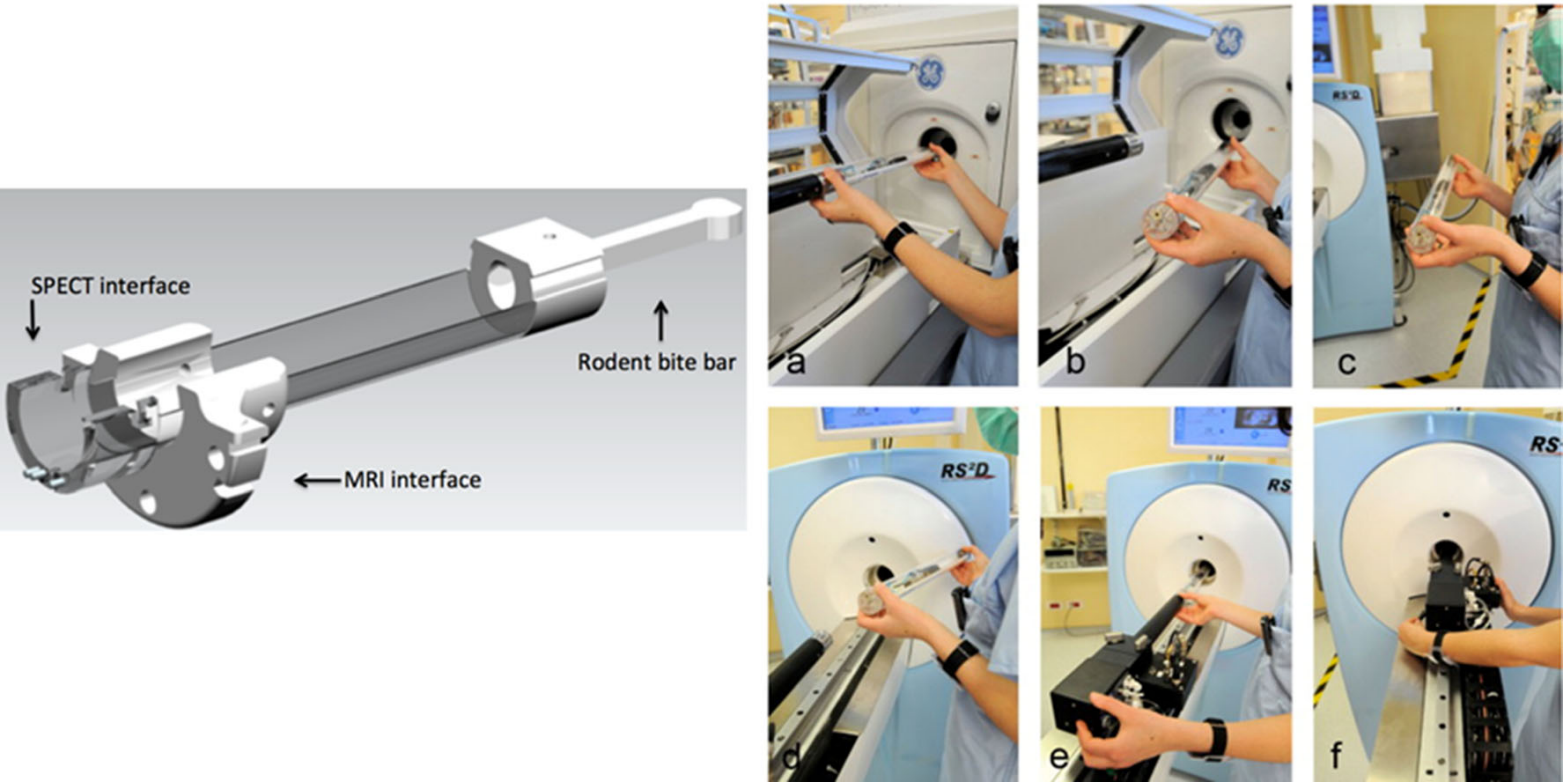
Example of the principle of a transferable bed system. *Left* Schematic drawing of an animal bed with tailored interfaces for mounting into compatible cradles in SPECT and MRI scanners. *Right* Step-by-step photo representation of the transfer from a SPECT scanner to a MRI scanner: **a** at the end of SPECT/CT acquisition; **b** the animal bed is unplugged; **c**, **d** the animal and bed are moved towards the MRI scanner ; **e**, **f** the bed is docked and positioned inside the magnet followed by MRI acquisition (image courtesy of Philippe Choquet)

In contrast to clinical imaging of patients, small animals can be transported – including the bed – between imaging devices with gentle fixation with tape preventing movement of the animal on the bed. This requires beds that can be easily, rigidly and reproducibly mounted on different scanners (Fig. 4). Multimodal fiducial markers attached to the animal (or bed) or a premeasured transformation matrix can be used for spatial coregistration [135, 136]. Such side-by-side use of separate scanners offers flexibility in adding and/or replacing individual modalities while both systems can be used in parallel facilitating higher through-put. However, maintaining anaesthesia may be a challenge during transport, especially when the machines are far apart.

#### In-line systems

**Fig. 5:**
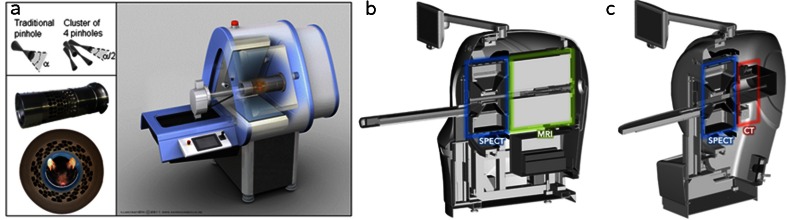
Combined modality approaches. **a** Drawing of a SPECT/CT system in which the SPECT part can also image 511 keV photons to perform simultaneous SPECT/PET (from M.C. Goorden et al., JNM 2013). **b**, **c** Cross-sectional views of (**b**) a proposed SPECT/MRI system and (**c**) a SPECT/CT system. For **b** and **c** the SPECT system is placed in front while the MRI or CT system is placed at the back of the scanner (**b**, **c** courtesy of Mediso Medical Imaging Systems)

A second approach to imaging with SPECT in combination with CT or MRI is to mount the separate modalities in line (i.e. back-to-back) on a single gantry (Fig. 5). When the bed moves in the axial direction, images of the different modalities can be acquired shortly after each other. With this approach it is easier to continuously provide anaesthesia and no animal handling between scans is required. However, simultaneous use of the separate modalities is not possible, limiting flexibility and through-put. Furthermore, close proximity of the SPECT and MRI systems limits the MRI field strengths that can be applied potentially resulting in impractically long MRI acquisition times. MRI-compliant SPECT hardware will most likely tackle these problems in the future.

#### Integrated systems

**Fig. 6:**
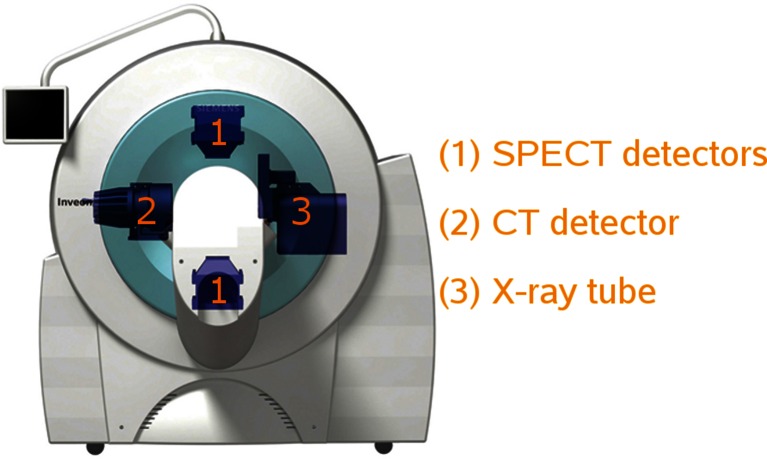
Diagram of an integrated SPECT/CT system showing two SPECT detectors, a CT detector and an X-ray tube, all rotating on the same gantry (image courtesy of Siemens Healthcare)

Figure 6 shows an example of a system where the SPECT and CT are mounted on the same gantry. An advantage is that fast sequential SPECT and CT acquisition can be performed with minimal or even without shifting of the bed. One of the drawbacks of this approach is space constraints, since the number and/or size of detectors that can be used for each modality is limited, preventing e.g. stationary and full angular SPECT approaches.

### SPECT combined with CT

Implementation of multipinhole collimators with high pinhole magnification factors in dedicated small-animal SPECT systems has helped overcome the limitation of poor sensitivity and spatial resolution. Efforts have been made to keep the heavy SPECT detectors stationary [16, 137–139] in order to obviate the need for regular geometric parameter calibration and to enable fast dynamic imaging [3, 137], while sensitivity and resolution in organ and tumour imaging have been increased [139–141].

CT systems currently used in preclinical SPECT/CT usually contain a variable energy X-ray tube. Tube voltage and current are in the range of 20 – 80 kVp and 0.2 – 1 mA, respectively. Tube current typically decreases with decreasing focal spot size. Reconstructed resolutions of well below 100 μm are achieved using microfocus X-ray tubes with focal spot sizes down to a few micrometres.

### SPECT combined with MRI

**Fig. 7:**
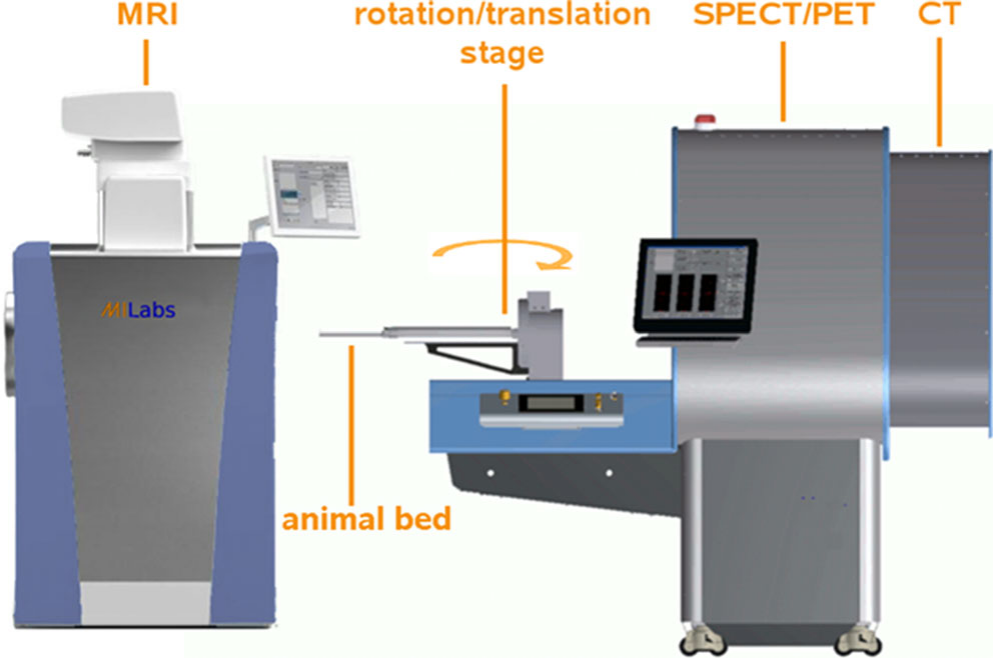
One of the commercial side-by-side solutions for integrating 1.5-T or 3-T MRI with SPECT and other modalities. In this example a robotic rotation/translation stage automatically transfers the animal between the systems. In this set-up the MRI system is integrated in line with the other modalities, while avoiding possible interference of the fringe magnetic field of the MRI system with the other modalities (image courtesy of MILabs B.V.)

Exposure to ionizing radiation from CT imaging may influence study outcomes [142–146]. Furthermore, image contrast of CT is often suboptimal for soft tissues such as brain and tumours. These two limitations have been strong incentives for the current efforts to integrate SPECT and MRI. A combined SPECT/MRI platform was first proposed in 2007 by Breton et al. who used a single pinhole SPECT system adjacent to a 0.1-T magnet [147]. The low MRI field strength made this solution suboptimal for use in routine preclinical research. However, since then systems combining SPECT and MRI have been introduced with both higher SPECT sensitivity and resolution and higher MRI field strengths. One solution involves the use of a robotic rotation/translation stage that automatically transfers the animal between the separately spaced MRI system and other modalities (Fig. 7). Using such an approach the MRI unit is still positioned in line with the other modalities, while avoiding the effects of fringe magnetic fields. Similar to hybrid SPECT/CT scanners, recent efforts also include an in-line hybrid SPECT/MRI system, in which the SPECT subsystem is placed in front of the MRI system (Fig. 5b). In attempts to perform simultaneous SPECT and MR imaging, SPECT inserts for MRI systems have been developed [148, 149]. They have a stationary detector set-up and MRI-compatible collimators and detectors, although today these systems are still in early stages of development.

### Quantification in animal SPECT combined with CT or MRI

Preclinical SPECT systems are mostly based on the use of pinholes that magnify projections of the radionuclide distribution on detectors. For proper quantification of radioactivity, image-degrading factors such as distance-dependent collimator response and sensitivity, as well as photon attenuation and scatter, need to be taken into account.

#### Distance-dependent collimator response and sensitivity

To maximize spatial resolution in SPECT, thereby minimizing partial volume effects, and to reduce quantification errors, distance-dependent collimator resolution and sensitivity need to be taken into account in image reconstruction (i.e. resolution recovery methods). In this context it is also important to accurately calibrate the system’s geometrical parameters [150–154]. Methods that also account for more complex effects, such as detector and collimator imperfections include measurements of the system’s response with a point source at many discrete locations in the field of view of the camera [155–157]. Such methods can also be combined with advanced interpolation schemes [158] and have enabled very high spatial resolution.

#### Attenuation and scatter

Since the likelihood of scatter events in small animals is much smaller than in humans, the effects of photon attenuation and scatter in tissue are smaller than in clinical SPECT [159]. Simulation studies in mouse-sized phantoms have shown that attenuation can degrade quantitation accuracy by up to −18 % (^99m^Tc or ^111^In) or −41 % (^125^I) [160]. Accounting for scatter and attenuation is especially important for imaging tracers that emit low gamma-ray energies such as ^125^I [161, 162]. Several methods have been published for attenuation and scatter correction [161, 163–166]. First order attenuation correction methods as proposed in 1978 by Chang [167] seem to be sufficient for small-animal SPECT [165]. Attenuation correction maps can be derived from CT images [161], optical images [165] and MR images [163].

Because of the low amount of scatter in small subjects, simple energy-window-based corrections [168–170] are often sufficient for ^99m^Tc, ^123^I and ^111^In [161, 165, 166]. However, in the case of multipeak spectra and multiradionuclide imaging, it is important that many scatter windows are available, or that data are acquired in list mode (i.e. that for each detected photon its position, its energy and its detection time are stored). Scatter in pinhole apertures is low [171], although in multienergy and in multiradionuclide SPECT, scatter and photon penetration in the collimator can be a significant issue, e.g. with a combination of SPECT and PET tracers used on a SPECT camera, although in such a case excellent quantitative images have been recently obtained using a dedicated high-energy (clustered-)pinhole collimator and window-based scatter corrections [14].

## Concluding remarks and future perspectives

Recent advances in small-animal SPECT/CT and SPECT/MRI devices, radiochemistry, probe development, target finding and suitable animal models have provided more advanced and increased applications of these combined imaging strategies.

In most preclinical SPECT imaging studies to date, CT or MRI merely fulfil a supportive role to provide anatomical reference and in some cases attenuation correction. In small laboratory animals, acquisition of detailed anatomical information, performance of dynamic scans or functional imaging with CT has specific challenges compared to imaging in humans. To reach diagnostic image quality high CT radiation doses and/or large volumes of contrast agent are necessary. These aspects are not compatible with longitudinal studies, since they may severely affect the wellbeing of animals. New developments in small-animal CT [172–177] and the use of new contrast agents for CT should provide better image quality at lower radiation doses and/or with lower volumes of contrast agent.

MRI offers detailed anatomical imaging of soft tissues compatible with longitudinal studies. Separately spaced SPECT and MRI systems can pose challenges with respect to image registration, imaging times and anaesthesia times; the effects of these issues can potentially influence study results. On the other hand, physical integration of SPECT and MRI technologies is hampered by various incompatibilities; the components and working mechanisms of the separate modalities currently degrade the other’s performance.

A major benefit of higher magnetic field strengths is that more signal is provided which can be used to shorten acquisition times, but higher magnetic field strengths do not always guarantee higher image quality per se. Currently, most high-field magnets are cooled with cryogenic liquids. One of the main drivers towards small MRI systems is the development of cryogen-free magnets or systems using helium gas instead of liquid helium. This development could lead to significant reductions in the size, site requirements and cost of high-field MRI systems. Today, such helium gas-based commercial MRI systems are limited to 3 T.

While not covered in detail in this review, an extremely important aspect to consider during imaging is animal welfare. Animal handling and especially anaesthesia is demanding to the animal and can severely affect the outcome of imaging studies [35, 178–180]. Also issues regarding radiation doses will have to be taken into account [142, 143, 181, 182].

Taking these issues into account, further advances in technology and chemistry, for example the development of new imaging procedures and promising new radiopharmaceuticals, for imaging molecular targets as well as (patho)physiological processes in vivo, the step from bench to bedside might become more successful and shorter; e.g. accelerating the introduction of new imaging procedures and promising new radiopharmaceuticals into clinical practice.
